# Hemilateral adipose and muscular atrophy associated with a somatic PDGFRB p.N666K variant

**DOI:** 10.1016/j.gendis.2026.102050

**Published:** 2026-01-22

**Authors:** Yuan-yang Zheng, Hongrui Chen, Bin Sun, Chen Hua, Xiaoxi Lin

**Affiliations:** Department of Plastic & Reconstructive Surgery, Shanghai Ninth People's Hospital, Shanghai Jiao Tong University School of Medicine, Shanghai 200011, China

PDGFRB encodes a transmembrane receptor tyrosine kinase that plays a central role in mesenchymal cell development and tissue homeostasis, particularly in vascular maturation, adipogenesis, and skeletal growth. Germline and somatic variants in PDGFRB have been identified in a spectrum of PDGFRB-related disorders, including myofibromatosis, fusiform aneurysms, Kosaki overgrowth syndrome, and Penttinen premature aging syndrome. These conditions are typically characterized by tissue overgrowth, vascular anomalies, or fibrotic lesions.^1^

Here, we report a novel clinical phenotype associated with a somatic PDGFRB variant, characterized by hemilateral adipose and muscular atrophy, accompanied by vascular lesions and a disproportionate arm span. To our knowledge, this is the first case of PDGFRB-related disease presenting predominantly with tissue atrophy rather than overgrowth, expanding the phenotypic spectrum of PDGFRB variants and highlighting the context-dependent roles of PDGFRB signaling in human tissue homeostasis.

A 13-year-old boy was referred for evaluation of congenital right-sided hemilateral atrophy, first noted at one month of age and progressing proportionally with growth. The asymmetry was characterized by visibly reduced girth of the right upper and lower limbs compared to the contralateral side, alongside diminished subcutaneous fat and muscle bulk. Prominent superficial veins and telangiectatic capillary networks were observed across the affected limbs and chest wall (Fig. 1A and B). On palpation, these lesions were soft, compressible, and without local warmth or pulsation, consistent with a low-flow vascular process. The parents reported no functional limitations, pain, or psychomotor developmental abnormalities. The patient, born to non-consanguineous parents with no family history of neuromuscular disorders or asymmetric growth, exhibited a slender stature with a height of 163.3 cm and an elevated arm span-to-height ratio of 1.11 (arm span: 181.2 cm).

**Figure 1:**
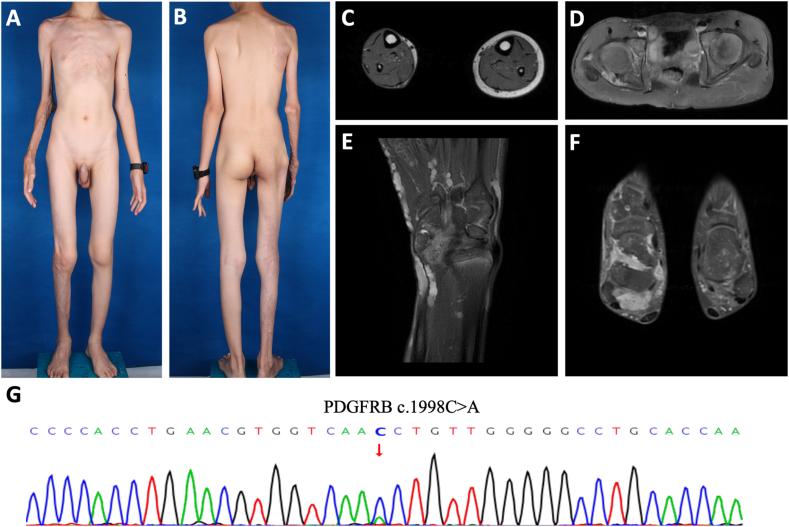
The patient's phenotypic and genetic features. **(A, B)** Anterior and posterior views of the patient showing right-sided hemilateral adipose and muscular atrophy, with prominent superficial veins and an increased arm span-to-height ratio. **(C)** Axial T1-weighted MRI showing reduced subcutaneous fat and muscle bulk in the right limbs. **(D**–**F)** MRI revealed multiple vascular lesions in the right limbs, presenting as heterogeneous enhancement on contrast-enhanced T1-weighted images (D, F) and high signal intensity on T2 fat-suppressed imaging (E). **(G)** Validation of the *PDGFRB* c.1998C>A (p.N666K) variant by Sanger sequencing.

Anthropometric measurements revealed minimal asymmetry in hand and foot lengths, though both hands appeared disproportionately large for his stature. Marked right-sided hemilateral atrophy was observed, with reduced circumferential measurements: the right mid-thigh measured 34.6 cm (left 37.2 cm, 7% reduction) and the right calf 23.0 cm (left 25.8 cm, 10.9% reduction). His facial features were normal, without dysmorphic characteristics suggestive of premature ageing or Kosaki overgrowth syndrome.

MRI of the lower limbs revealed a marked reduction in subcutaneous fat and muscle bulk on the right side compared to the left (Fig. 1C), consistent with the clinical findings of hemilateral adipose and muscular atrophy. Imaging also revealed multiple vascular lesions in the subcutaneous tissue, intermuscular spaces, and periarticular regions of the right upper and lower limbs (Fig. 1D–F), but no neurological deficits or systemic abnormalities were observed.

Targeted sequencing identified a somatic PDGFRB c.1998C>A (p.N666K) missense variant in the affected subcutaneous tissue, with a mosaic variant allele frequency of approximately 21.17%. Notably, this variant was not reported in the gnomAD database in either the general population or the East Asian subpopulation, suggesting its rarity and potential pathogenicity. Sanger sequencing confirmed the presence of this variant in the lesion tissue but not in the peripheral blood, indicating its somatic origin (Fig. 1G).

PDGFRB signaling is essential for pericyte recruitment during angiogenesis and has been shown to inhibit adipocyte differentiation in a cell-autonomous manner in murine models.^2^ The identified p.N666K variant is located within the tyrosine kinase domain of PDGFRB (residues 600–962), a region essential for receptor activation and downstream signaling. This variant has been previously reported in the somatic state in multiple patients with infantile myofibromatosis (IM).^1^ Structural modeling suggested that the p.N666K substitution disrupts the interaction between Asn666 and His661 within the N-terminal lobe of the kinase domain, potentially leading to an active conformation of the receptor. This mutation leads to constitutive activation of downstream signaling pathways, driving cell proliferation and survival, and has been shown to confer sensitivity to tyrosine kinase inhibitors. Variants affecting the same residue (Asn666) have been recurrently identified in a spectrum of PDGFRB-related disorders, such as Penttinen syndrome (p.N666S), and Kosaki-Penttinen overlapping syndrome (p.N666H), highlighting the functional importance of this hotspot.^1^

The mosaic expression of the p.N666K variant provides a plausible explanation for the patient's focal and unilateral phenotype. Somatic activation of PDGFRB in the affected tissues may have led to abnormal vascular proliferation and impaired adipogenesis or muscle development, resulting in localized atrophy and vascular lesions. The absence of systemic involvement and the confinement of clinical manifestations to one side of the body further support a postzygotic mutational event.

Although activating PDGFRB variants have been associated with several syndromes, the phenotype observed in this patient is distinct. Muscle atrophy has not been previously linked to PDGFRB variants. In this patient, several mechanisms may underlie the observed muscular involvement. MRI demonstrated vascular lesions within intramuscular compartments (Fig. 1D), suggesting secondary ischemic or degenerative changes due to altered local perfusion. Moreover, as PDGFRβ is a key receptor in pericyte and vascular smooth muscle cell development, the variant may disrupt vessel stabilization and local hemodynamics. Notably, PDGFRβ-expressing pericytes play a critical role in skeletal muscle regeneration^3^; aberrant PDGFRβ signaling may compromise this function, resulting in muscle atrophy instead of effective regeneration. However, whether the PDGFRB variant directly affects myogenic development or muscle homeostasis remains to be clarified in future studies.

Clinically observed telangiectasias and MRI findings in this case indicated low-flow vascular malformations without aneurysmal features, distinct from the segmental capillary malformations and AVM/AVF reported in mosaic *PDGFRB* p.Tyr562Cys variants.^4^^,^^5^ There was no evidence of myofibromas. While Penttinen syndrome is characterized by lipoatrophy and prominent veins, our patient did not display its hallmark features, such as premature aging, acro-osteolysis, or ocular adhesions. Similarly, Kosaki overgrowth syndrome was excluded based on the absence of overgrowth, dysmorphic facial features, white matter lesions, or neurological deterioration. Moreover, skeletal abnormalities commonly reported in PDGFRB-related disorders, such as scoliosis or irregular skull contour, were not present.^1^ Taken together, this case represents a novel, localized phenotype expanding the clinical spectrum of PDGFRB variants.

Our findings highlight the importance of considering somatic mosaicism in patients with segmental or asymmetric phenotypes. This case expands the phenotypic spectrum of PDGFRB-related disorders and suggests that activating PDGFRB mutations may lead to highly tissue-specific manifestations depending on their mosaic distribution and the local cellular context. Given the pleiotropic roles of PDGFRB signaling in regulating mesenchymal cell behavior, vascular stability, adipogenesis, and skeletal development, its dysregulation may exert distinct biological effects in different tissue environments. These observations underscore the context-dependent roles of PDGFRB in maintaining human tissue homeostasis and remodeling. Tyrosine kinase inhibitors, such as imatinib, have shown therapeutic potential in PDGFRB-driven diseases by blocking aberrant receptor signaling. However, the long-term efficacy and safety of such targeted therapies, particularly in mosaic or tissue-restricted conditions, remain to be determined. Future studies are warranted to better characterize the natural history of PDGFRB-related mosaic disorders and to explore personalized therapeutic strategies based on the distribution and functional impact of somatic mutations.

## CRediT authorship contribution statement

**Yuan-yang Zheng:** Writing – original draft. **Hongrui Chen:** Resources, Formal analysis. **Bin Sun:** Supervision, Investigation. **Chen Hua:** Writing – review & editing, Conceptualization. **Xiaoxi Lin:** Writing – review & editing.

## Ethics declaration

This study was approved by the Ethics Board of Shanghai Ninth Hospital, Shanghai Jiaotong University of Medicine (No. SH9H-2021-C46). Diagnostic genetic testing was performed after written informed consent was received from the patients' parents, in accordance with local regulations. Written informed consent was obtained from both patients’ parents for the publication of clinical information and patient photographs.

## Funding

This work was supported by Fundamental research program funding of Ninth People's Hospital affiliated to 10.13039/501100008233Shanghai Jiao Tong University School of Medicine (JYZZ241), the Top Priority Research Center of Shanghai-Plastic Surgery Research Center, China (No.2023ZZ02023), the 10.13039/501100012226Fundamental Research Funds for the Central Universities of China (No.YG2023ZD13), and the Treatment and Mechanism of PI3K/mTOR Dual-Target Inhibitor (WX390) on PIK3CA-Related Overgrowth Spectrum (PROS) (China) (No. JYHX2022004).

## Conflict of interests

All authors declare that there are no competing interests.
