# Supplementary material for: Hemilateral adipose and muscular atrophy associated with a somatic PDGFRB p.N666K variant
Source: Genes Dis. 2026 Jan 22;13(6):102050. doi: 10.1016/j.gendis.2026.102050 (PMC13380725; doi:10.1016/j.gendis.2026.102050)
Supplement: Multimedia component 1 [file mmc1.docx]

**Hemilateral Adipose and Muscular Atrophy Associated with a Somatic PDGFRB p.N666K Variant**

**Materials & Methods**

**DNA extraction and next-generation sequencing (NGS)**

DNA was extracted from peripheral blood samples and lesion tissue biopsies obtained from the patient. Targeted sequencing of 44 genes associated with cancer and developmental disorders was performed using the DNBSEQ-T7 platform (MGI, China) and the xGen Exome Research Panel v1.0 (Integrated DNA Technologies, Inc., USA). This approach generated an average sequencing output of 3,389.30 megabases per sample, with a mean coverage of 12,051× across the targeted regions.100% of the regions were covered by ≥10 reads and ≥30 reads, ensuring high-confidence variant calling. Sequencing reads were aligned to the human reference genome (hg19) using the Burrows-Wheeler Aligner (BWA), followed by local realignment around indels and base quality score recalibration using the Genome Analysis Toolkit (GATK v3.7).

**Sanger sequencing**

PCR amplification was performed using a Veriti 96-Well Thermal Cycler (Applied Biosystems, Thermo Fisher Scientific, MA) with 2× Taq Plus Master Mix (Vazyme Biotech, P211) according to the manufacturer’s instructions. PCR products were analyzed by Sanger sequencing using a 3730 DNA Analyzer (Applied Biosystems, Thermo Fisher Scientific, MA) at We-health (Shanghai, China). Sequence data were analyzed using CodonCode Aligner software (CodonCode Corporation, USA).
